# Characteristics and potential biomarkers of flavor compounds in four Chinese indigenous chicken breeds

**DOI:** 10.3389/fnut.2023.1279141

**Published:** 2023-10-12

**Authors:** Xinwei Xiong, Jinge Ma, Qin He, Xiaolian Chen, Zhangfeng Wang, Longyun Li, Jiguo Xu, Jinfang Xie, Yousheng Rao

**Affiliations:** ^1^Key Laboratory for Genetic Improvement of Indigenous Chicken Breeds of Jiangxi Province, Nanchang Normal University, Nanchang, China; ^2^Institute of Animal Husbandry and Veterinary Medicine, Jiangxi Academy of Agricultural Sciences, Nanchang, China

**Keywords:** Chinese indigenous chicken, meat flavor, volatile organic compounds, potential biomarkers, breeds

## Abstract

Chinese indigenous chickens have a long history of natural and artificial selection and are popular for their excellent meat quality and unique flavor. This study investigated six meat quality-related traits in Ningdu yellow, Baier yellow, Kangle, and Shengze 901 chickens. Two-dimensional gas chromatography-time-of-flight mass spectrometry was used to detect unique flavors in 24 breast muscle samples from the same phenotyped chickens. Overall, 685, 618, 502, and 487 volatile organic compounds were identified in Ningdu yellow, Baier yellow, Kangle, and Shengze 901 chickens, respectively. The flavor components were separated into eight categories, including hydrocarbons and aldehydes. Multivariate analyses of the identified flavor components revealed some outstanding features of these breeds. For example, the hydrocarbons (22.09%) and aldehydes (14.76%) were higher in Ningdu yellow chickens and the highest content of N, N-dimethyl-methylamine was in Ningdu yellow, Baier yellow, and Shengze 901 chickens, indicating the maximum attribution to the overall flavor (ROAV = 439.57, 289.21, and 422.80). Furthermore, we found that 27 flavor compounds differed significantly among the four Chinese breeds, including 20 (e.g., 1-octen-3-ol), two (e.g., 2-methyl-naphthalene), four (e.g., 2,6-lutidine), and one (benzophenone) flavor components were showed significant enrichment in Ningdu yellow, Baier yellow, Kangle, and Shengze 901 chickens, respectively. The flavor components enriched in each breed were key biomarkers distinguishing breeds and most were significantly correlated with meat quality trait phenotypes. These results provide novel insights into indigenous Chinese chicken meat flavors.

## 1. Introduction

Chicken is an important poultry species in the meat industry and is distributed worldwide. The main meat quality traits include meat color, drip loss, and pH, which have been improved over the past 20 years with the adaptation of novel genomic techniques. Moreover, meat flavor is a crucial factor for consumers when evaluating and accepting meat (1). All these traits are complex and are affected by multiple factors, such as breed. An increasing number of studies have found that the flavor of most indigenous chicken breeds is associated with excellent meat quality and a high nutritional value (1, 2). Therefore, it is important to understand the scientific basis of the unique flavors of different chicken breeds, especially indigenous breeds, to improve chicken meat quality and to satisfy consumer requirements.

Flavors are more effective than taste and texture at distinguishing important quality characteristics of meat products. The meat flavor is primarily determined by volatile organic compounds (VOCs), which are mainly produced by the Maillard reaction and lipid oxidation between lipids and water-soluble organic compounds (3, 4). Flavor compound analysis techniques include two-dimensional gas chromatography-time-of-flight mass spectrometry (GC × GC-TOF-MS), gas chromatography-ion mobility spectrometry (GC-IMS), gas chromatography–olfactometry-mass spectrometry (GC-O-MS), and gas chromatography–mass spectrometry (GC–MS). Yang et al. identified 89 flavor components in samples from six commercial smoked chicken products in China using GC–MS (5). Benzothiazole, 2-Pyrrolidinone, 2-Acetylthiazole, and disulfide dipropyl have been identified in Dezhou braised chickens using GC × GC/HR-TOFMS (6). Zhou et al. investigated flavor changes in Fuliji roast chicken during processing and storage times based on GC-IMS and identified 47 VOCs across seven sampling stages (2). Furthermore, Kamatou et al. identified more VOCs, especially fatty acid methyl esters, in palm and palmist oils than in GC × GC-TOF-MS and GC–MS, (7). Li et al. investigated VOCs in six Chinese dry-cured hams and found that GC × GC-TOF-MS detected more than five times more VOCs than GC-IMS (8). Therefore, a comprehensive flavor component study using GC × GC-TOF-MS is required to better understand unique flavor formation in indigenous Chinese chicken meat.

Ningdu yellow, Baier yellow, and Kangle chickens are representative Chinese indigenous breeds of yellow-feathered broilers. These breeds were developed through natural and artificial selection processes and are known for their distinct and unique flavors. As a representative cultivated variety of white-feathered broiler breeds, Shengze 901 chickens have faster growth rates and lower breeding costs. White-and yellow-feathered broilers are the dominant breeds in the Chinese chicken production industry. In this study, to investigate the unique flavor formation in Chinese broiler breeds, GC × GC-TOF-MS was performed on breast muscle harvested from Chinese Ningdu yellow, Baier yellow, Kangle, and Shengze 901 chickens. The main and unique flavor compounds of four Chinese breeds were identified and comparisons between the white-and yellow-feathered broilers were performed by multivariate analyses, including Venn diagram, relative odor activity value (ROAV), and FlavorDB analyses. Furthermore, comparative analysis and correlation analysis were used to fully understand the flavor differences among four Chinese breeds and the relationship with meat quality traits. Our results help identify unique flavors and provide a theoretical reference for improving chicken quality in the future.

## 2. Materials and methods

### 2.1. Ethics statement

All procedures involving animals followed the guidelines for the care and use of experimental animals as approved by the State Council of the People’s Republic of China. This study was approved by the ethics committee of Nanchang Normal University (No. NCNU2021-006).

### 2.2. Phenotype measurement and sample collection

Overall, 24 chickens were included in this study. Six each of Ningdu yellow, Baier yellow, and Kangle chickens were acquired from Jiangxi Nanshi Science and Technology Co., Ltd. (Nanchang, China), and six Shengze 901 chickens were acquired from the Jiangxi Academy of Agricultural Sciences (Nanchang, China). Under a standardized management and feeding regimen, all experimental chickens were fed the same diet (Supplementary Table S1) and were given free access to water. Ningdu yellow, Baier yellow, and Kangle chickens were slaughtered at the age of 150 days, while Shengze 901 chickens were slaughtered at the age of 42 days. After 12 h of overnight fasting, all 24 chickens were electrically stunned and then killed by exsanguination. The breast muscle tissues were placed into sterile and frozen cryopreservation tubes, dipped into liquid nitrogen, and then stored in an −80°C ultra-freezer. Furthermore, six meat quality traits were measured, including pH, L* for lightness (ColorM_L), a* for redness (ColorM_a), b* for yellowness (ColorM_b), moisture content, and intramuscular fat (IMF). The methods used to measure pH, ColorM_L, ColorM_a, and ColorM_b were described in our previous study (9). Each sample was measured thrice by the same researcher. The routine oven drying method was used to measure moisture content in duplicate (10). The Soxhlet extraction method was used to determine the IMF content in duplicate (11). Briefly, the packaged sample was placed into a Soxhlet extractor and 60–100 mL of anhydrous ether was added. The sample was heated in a 60–75°C water bath, the cooling tap was turned on, and the ether reflux rate was maintained at about 10 times per hour for 6–8 h. Finally, the weight difference before and after sample extraction was calculated. The average of each trait measurement was used for subsequent analyses.

### 2.3. GC × GC-TOF-MS analysis parameter

All 24 breast muscle samples were used for GC × GC-TOF-MS analysis. First, 2 g breast muscle was transferred into a 15 mL centrifuge tube which was placed into a foam box. Next, the appropriate liquid nitrogen to freeze for 5 min was added. The lyophilized sample was broken up in the crusher for 3 min, and then transferred into a 20 mL headspace vial with the addition of 10 μL of the internal standard solution, and then incubated for 10 min at 80°C. The solid phase microextraction (SPME) fiber was placed in a chamber at 270°C for 10 min before the sample was extracted and the SPME was transferred to an incubator at 80°C for 40 min. The SPME fiber was then desorbed at 250°C for 5 min in the GC injector after which it was put in the chamber at 270°C for 10 min. The GC × GC analysis was performed using a LECO Pegasus^®^ 4D instrument (LECO, St. Joseph, MI, United States) consisting of an Agilent 8890A GC (Agilent Technologies, Palo Alto, CA, United States) system equipped with a split/splitless injector, and dual stage cryogenic modulator (LECO) coupled with TOF MS detector (LECO). The DB-Heavy Wax (30 m × 250 μm I.D., 0.5 μm) (Agilent, United States) and Rxi-5Sil MS (2.0 m × 150 μm I.D., 0.15 μm) (Restek, United States) were used as the first-dimension column (1D) and second-dimension column (2D), respectively. High-purity helium (>99.999%) was used as the carrier gas at a constant flow rate of 1.0 mL/min. The temperature program of the oven was as follows: first, the oven temperature was held at 50°C for 4 min; secondly, the oven temperature was raised to 150°C at a rate of 3°C /min and held for 1 min; third, the oven temperature was raised to 200°C at a rate of 10°C /min and held for 0 min; finally, the oven temperature was increased to 220°C at a rate of 5°C /min and held for 10 min. The temperature of the modulator was always 15°C higher than that in the second column. The modulator was operated with a 6.0 s modulation period. The GC injector temperature was at 250°C. The flavoring substances were prepared using LECO Pegasus BT 4D. The temperatures of TOF MS ion source and transfer line were set to 250°C and the acquisition frequency was 200 spectra/s. In addition, the mass spectrometer was operated in the EI mode at 70 eV using a range of m/z 35–550 and a detector voltage of 1984 V. The GC × GC-TOF-MS analysis was performed as described by Song et al. (12).

### 2.4. Statistical analysis

Orthonormal partial least squares discriminant analysis (OPLS-DA), partial least squares discriminant analysis (PLS-DA), and principal component analysis (PCA) were performed to evaluate chicken breast samples and isolate the volatile organic compounds responsible for the differences among the four Chinese breeds (13). To determine the variations in abundance among four Chinese broiler breeds, a linear discriminant analysis (LDA) and effect size (LEfSe) analysis was performed. The analysis was performed with a significance level of α = 0.01, and an LDA score threshold of at least 2.50 (14). A random forest analysis was used to determine the flavor compounds that could be used to distinguish the breeds of Chinese chickens. The optimal number of flavor compounds was determined via 10-fold cross-validation using the random forest cross-validation function in the random forest package (15). To assess the relationship between differential flavor compounds and meat quality traits, spearman’s correlation analysis was used.

## 3. Results and discussion

### 3.1. Summary statistics and comparative analysis for meat quality traits

In this study, four Chinese chicken breeds were included (Supplementary Figure S1). Ningdu yellow chicken has the appearance characteristics of “three yellow” and “five red,” as well as the quality characteristics of “tender meat, delicious taste, and rich nutrition.” Baier yellow chicken derives its appearance from the criteria of “three yellows and one white,” with yellow feathers, yellow beak, yellow feet, and white ears. The main characteristics of Kangle chicken are yellow beak, feet, and fur, with a golden body color. Shengze 901 chicken is characterized by a faster growth rate, higher survival rate, higher egg production rate, and lower breeding cost. We phenotyped six each of Ningdu yellow, six Baier yellow, six Kangle, and six Shengze 901 chickens across six meat quality traits. The summarized statistics for the six meat quality traits are presented in Table 1. According to the coefficients of variation, we found that the ColorM_a and IMF showed great variation (>20%) in all breeds, with the coefficients of variation of ColorM_a ranging from 30.05 to 89.39%. In contrast, the coefficients of variation for pH and moisture content were relatively small (<2%) for all breeds. The coefficients of variation obtained here were similar to those reported in our previous large-scale study on Ningdu yellow chickens (9), which may be due to the fact that the causative genes and mutations were controlled ColorM_a and IMF in Ningdu yellow chickens.

**Table 1:** Summary statistics of meat quality traits from four Chinese breeds.

	Ningdu yellow chickens			Kangle chickens			Baier yellow chickens			Shengze 901 chickens		
Traits	Mean	S.D.^d^	C.V^e^ (%)	Mean	S.D.	C.V (%)	Mean	S.D.	C.V (%)	Mean	S.D.	C.V (%)
pH	5.63^c^	0.03	0.60	5.69^bc^	0.04	0.69	5.83^a^	0.07	1.16	5.78^ab^	0.10	1.67
ColorM_L^f^	53.62^a^	4.37	8.16	52.26^a^	3.72	7.13	45.66^bc^	2.98	6.53	45.58^c^	1.80	3.94
ColorM_a^f^	1.50^a^	1.13	75.17	2.31^a^	0.69	30.05	2.95^a^	2.64	89.39	3.63^a^	1.91	52.62
ColorM_b^f^	8.92^a^	1.67	18.72	9.52^a^	1.22	12.80	9.34^a^	1.78	19.02	10.69^a^	1.64	15.30
Moisture content	0.72^a^	0.01	1.50	0.73^a^	0.01	0.83	0.73^a^	0.01	1.29	0.74^a^	0.01	1.31
IMF^f^	1.92^a^	0.99	51.57	1.02^b^	0.28	27.75	0.73^b^	0.16	21.70	0.58^b^	0.15	26.50

^a–c^ Least-square means with different superscripts in the same row differ significantly (*p* < 0.05).

d Standard deviation.

e Coefficient of variation.

f ColorM_L, L* for lightness; ColorM_a, a* for redness; ColorM_b, b* for yellowness; IMF, intramuscular fat.

Next, we compared the differences in Chinese chicken meat quality traits among four different breeds. ColorM_a, ColorM_b, and moisture content did not differ significantly between each of the four breeds. Baier yellow chickens had the highest average pH (5.83), whereas Ningdu yellow chickens had the lowest pH (5.63). The pH was lower (*p* < 0.05) in Ningdu yellow and Kangle chickens than that in Baier yellow chickens. In contrast to pH, the ColorM_L value was higher (*p* < 0.05) in Ningdu yellow and Kangle chickens than that in Baier yellow chickens. Furthermore, Ningdu yellow chickens had the highest average IMF (1.92) which was significantly higher (*p* < 0.05) than that in the Kangle, Baier yellow, and Shengze 901 chickens. These results indicate that there are many differences in meat quality traits among Chinese chicken breeds. Furthermore, the breed is one of the most crucial factors influencing meat quality traits (16).

### 3.2. Three-dimensional plots of chicken meat from four Chinese breeds by GC × GC-TOF-MS

**Figure 1:**
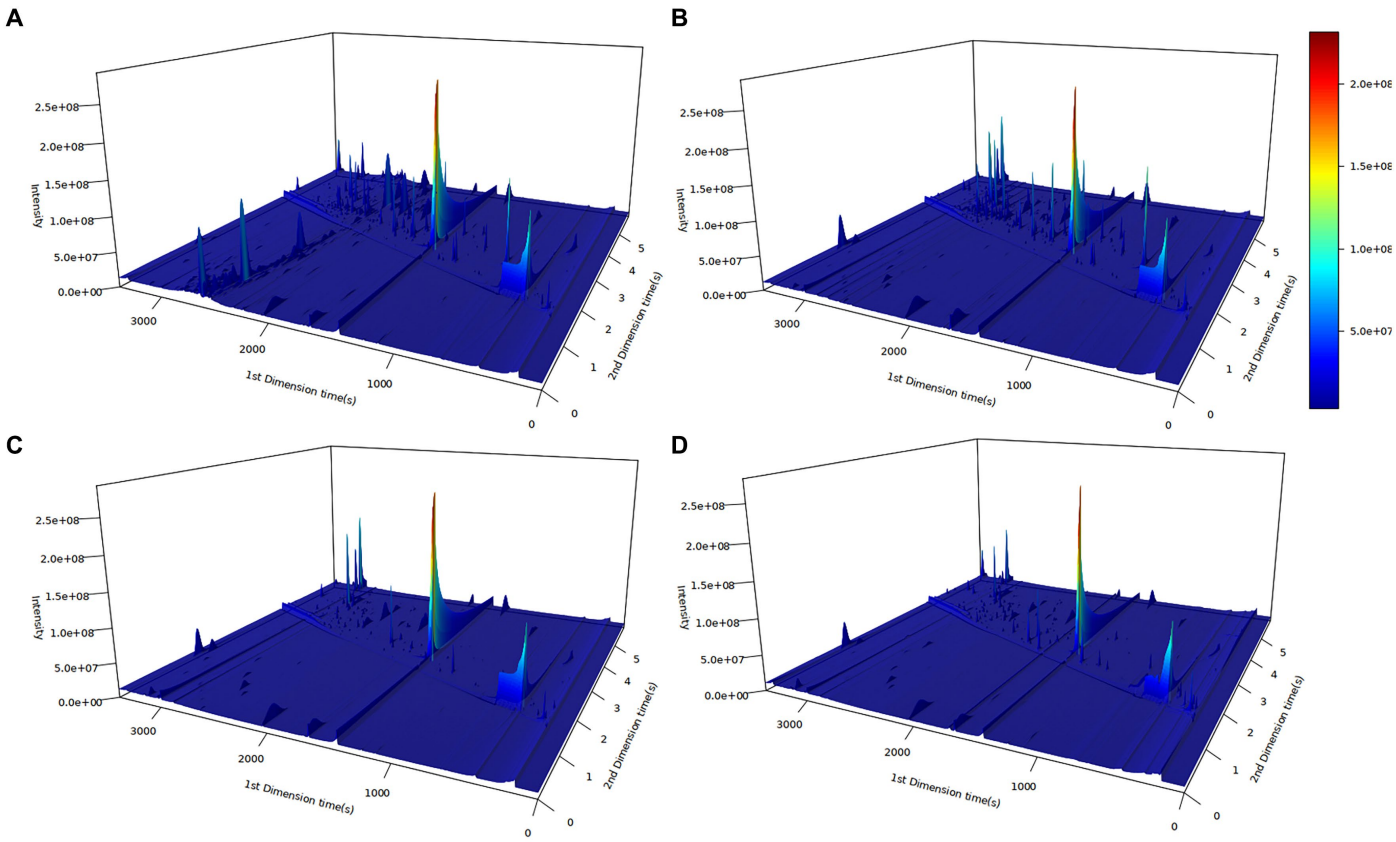
Three-dimensional (3D) plots of chicken meat from four Chinese breeds by GC × GC-TOF-MS. **(A)** Ningdu yellow chicken, **(B)** Baier yellow chicken, **(C)** Kangle chicken, **(D)** Shengze 901 chicken. The vertical and horizontal coordinates represent the one- and two-dimensional retention time, respectively. The color and peak height indicate the ion response intensity. Deeper red colors indicate higher response intensity.

The principles and applications of GC × GC-TOF-MS have been addressed in previous studies (8, 12, 17). In this study, GC × GC-TOF-MS was performed to obtain information on volatile organic compounds in chicken meat samples to identify differences in flavor substances among different chicken breeds. The results were visualized in the form of 3D chromatograms (Figure 1). Differences between volatile organic compounds in meat samples from different chicken breeds were observed due to the differences in natural or artificial selection in different breeds, resulting in differences in the flavor compounds of chicken meat.

### 3.3. The characteristic analysis of flavor compounds obtained by GC × GC-TOF-MS

**Figure 2:**
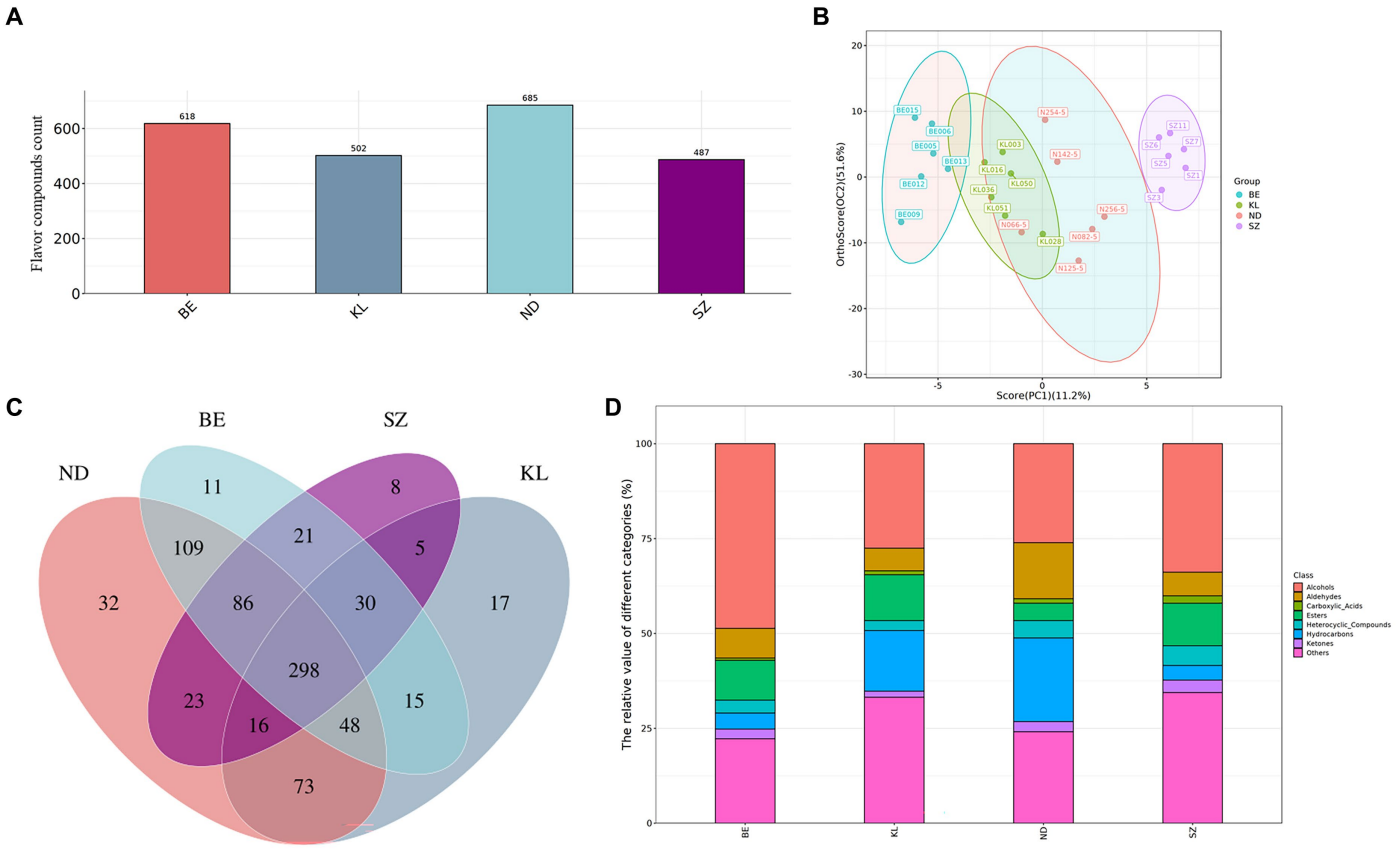
The characteristic analysis of volatile compounds in chicken meat from four Chinese breeds. **(A)** The number of volatile components identified in four Chinese chicken breeds. **(B)** OPLS-DA plot in samples from four Chinese chicken breeds. **(C)** Venn diagrams showed specifically volatile components of the four Chinese chicken breeds. **(D)** The relative values of different metabolite categories. BE, Baier yellow chicken; KL, Kangle chicken; ND, Ningdu yellow chicken; SZ, Shengze 901 chicken.

Based on the GC × GC-TOF-MS retention time and signal intensity, qualitative and quantitative analyses of the volatile organic compounds in chicken meat were performed for the four different breeds. In total, 791 flavor compounds were identified in all 24 chicken meat samples, including 685 from Ningdu yellow, 618 from Baier yellow, 502 from Kangle, and 487 from Shengze 901 chickens (Figure 2A). The PCA (Supplementary Figure S2) and OPLS-DA (Figure 2B) were used to analyze the differences in flavor compounds of breast muscle from the four Chinese chicken breeds. Because PCA and OPLS-DA can highlight interrelationships between different samples and simplify data, they are usually utilized as multivariate statistical tools for sample variation analyses (18, 19). In this study, the total contribution rate of the first and second components was 62.8% in OPLS-DA analysis, including the contribution rate of PC1 (11.2%) and PC2 (51.6%). The results indicated that the total contribution rates of PC1 and PC2 represented the main characteristics of the breast muscle samples from the four Chinese broiler breeds. This result was the same as for other species. For example, Ruan et al. found that the total contribution rate of the first and second components was 69.9% and that there was a significant difference in the aroma of fragrant rice between inorganic and organic cultivations (20). In the present study, the OPLS-DA results also showed that the volatile components exhibited an obvious shift among the four Chinese broiler breeds. Venn diagrams were used to identify the flavor compounds of the four breeds. The results showed that 32, 11, 17, and 8 flavor compounds were specifically identified in the Ningdu yellow, Baier yellow, Kangle, and Shengze 901 chickens, respectively (Figure 2C). In particular, compared to Shengze 901 chickens, 48 flavor compounds, such as 5-methyl-2-heptene, N-methyltaurine, and 3-methyl-undecane, were identified in only three indigenous Chinese broiler breeds (Ningdu yellow, Baier yellow, and Kangle chickens). The results also indicated that there are many differences in flavor between the white-and yellow-feathered broilers.

Finally, all the flavor compounds were separated into eight categories: ketones, hydrocarbons, heterocyclic compounds, aldehydes, esters, alcohols, carboxylic acids, and others (Supplementary Figure S3). The relative values of different metabolite categories are shown in Figure 2D. Alcohols were the main volatile flavor compounds in the four Chinese breeds of chicken breast muscle. Alcohols are mainly produced by the degradation of linoleic acid in muscles by lipoxygenases and peroxidases, which contribute to the formation of an ideal flavor and aroma in meat and meat products (2, 21–23). Alcohols (48.64%) were higher in Baier yellow chickens than in other breeds. Furthermore, hydrocarbons (22.09%) and aldehydes (14.76%) were higher in Ningdu yellow chickens, esters (12.10%) were higher in Kangle chickens, and carboxylic acids (1.92%), heterocyclic compounds (5.18%), and ketones (3.29%) were higher in Shengze 901 chickens. The oxidation of aldehydes produces acids, and the reaction of acids with alcohols produces ethyl propionate, butyl butyrate, and other esters. Esters are usually synthesized by esterification reactions between acids resulting from the degradation of proteins, fats, and alcohols, or by transesterification (alcoholysis) of fatty acids in triglycerides and ethanol (21).

The contribution of flavor compounds to the overall flavor was evaluated by measuring the proportion of key flavor compounds among the total flavor compounds of chicken breast muscle and their corresponding sensory thresholds (24). The key odor compounds are defined as flavor compounds with ROAV ≥1, and the larger the ROAV, the greater the contribution of the substance to the flavor (25). The ROAV results are presented in Supplementary Figure S4A; Supplementary Table S2. ROAV values of 20, 17, 12, and 18 were ≥ 1 in Ningdu yellow, Baier yellow, Kangle, and Shengze 901 chickens, respectively. The high content of N, N-dimethyl-methylamine in Ningdu yellow, Baier yellow, and Shengze 901 chickens indicated the maximum contribution to the overall flavor (ROAV = 439.57, 289.21, and 422.80). The ROAV of 2,3-butanedione was highest in Kangle chickens. The ROAV of dimethyl sulfide was higher in Shengze 901 chickens than in three indigenous Chinese broiler breeds (Ningdu yellow, Baier yellow, and Kangle chickens). Furthermore, we used FlavorDB (26) to analyze and compare the sensory flavors of the substance (Supplementary Figure S4B). Compared with other breeds, the Ningdu yellow chickens had higher citrus, Baier yellow chickens had a higher fatty aroma, Kangle chickens had a higher fresh scent, and Shengze 901 chickens had higher green and fruity characteristics. The sweet, bitter, waxy, and floral flavors of the chicken meat were similar among the four Chinese broiler breeds.

### 3.4. The potential biomarkers of flavor compounds in chicken breast muscle from four Chinese breeds

**Figure 3:**
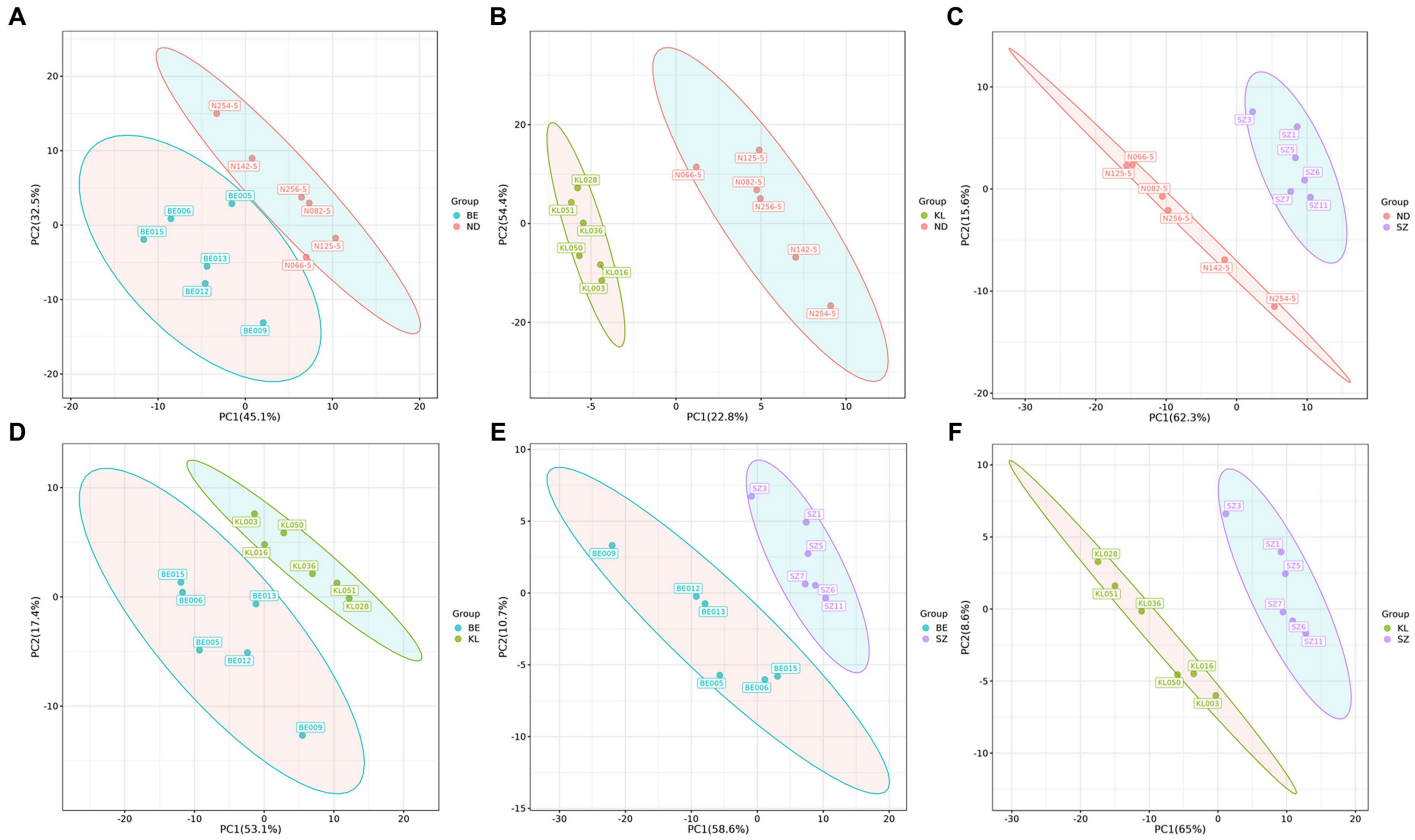
PLS-DA showed that the volatile compounds exhibited an obvious shift between any two of the four Chinese broiler breeds. **(A)** Ningdu yellow and Baier yellow chickens, **(B)** Ningdu yellow and Kangle chickens, **(C)** Ningdu yellow and Shengze 901 chickens, **(D)** Baier yellow and Kangle chickens, **(E)** Baier yellow and Shengze 901 chickens, **(F)** Kangle and Shengze 901 chickens. BE, Baier yellow chicken; KL, Kangle chicken; ND, Ningdu yellow chicken; SZ, Shengze 901 chicken.

**Figure 4:**
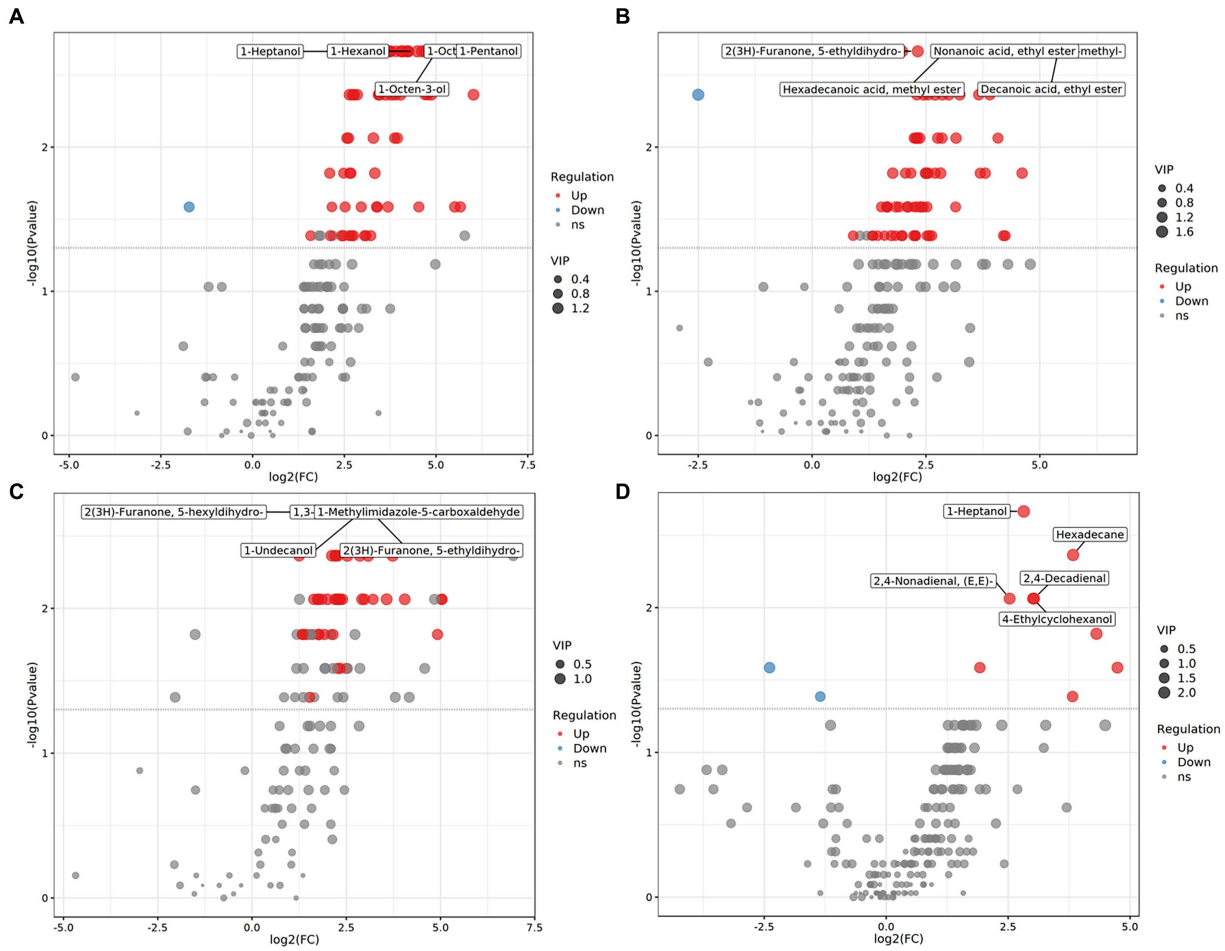
Volatile compounds showed distinct enrichment patterns among four Chinese chicken breeds. **(A)** Ningdu yellow and Shengze 901 chickens, **(B)** Baier yellow and Shengze 901 chickens, **(C)** Kangle and Shengze 901 chickens, **(D)** Ningdu yellow and Baier yellow chickens. The red dots represent upregulated differential volatile compounds, blue dots represent downregulated differential volatile compounds, and gray dots represent detected but not significantly different volatile compounds.

We systematically evaluated the shifts in flavor compounds in chicken breast muscle between any two of the four Chinese breeds. PLS-DA confirmed that the flavor compounds exhibited an obvious shift between any two of the four Chinese broiler breeds (Figure 3). Among the flavor compounds with statistical significance (*p* < 0.05, and VIP > 1), we identified 74, 67, and 98 flavor compounds showing distinct enrichment patterns between any one of the three indigenous Chinese breeds and Shengze 901 chickens (Supplementary Figure S5). For example, 73 compounds were increased in Ningdu yellow chickens and one compound was decreased as compared to Shengze 901 chickens (Figure 4A). A total of 66 compounds were increased in Baier yellow chickens and one compound was decreased compared to Shengze 901 chickens (Figure 4B). In comparison to Shengze 901 chickens, 98 flavor compounds were increased in Kangle chickens and none were decreased (Figure 4C). Furthermore, the number of flavor compounds was lower in any of the two breeds in the three indigenous Chinese breeds than in any one of the three indigenous Chinese breeds and Shengze 901 chickens (Supplementary Figure S5). Such as, nine compounds were increased in Ningdu yellow chickens and two compounds were decreased as compared to Baier yellow chickens (Figure 4D). These results indicate that different chicken breeds have unique meat flavor compositions, especially in indigenous Chinese breeds.

**Figure 5:**
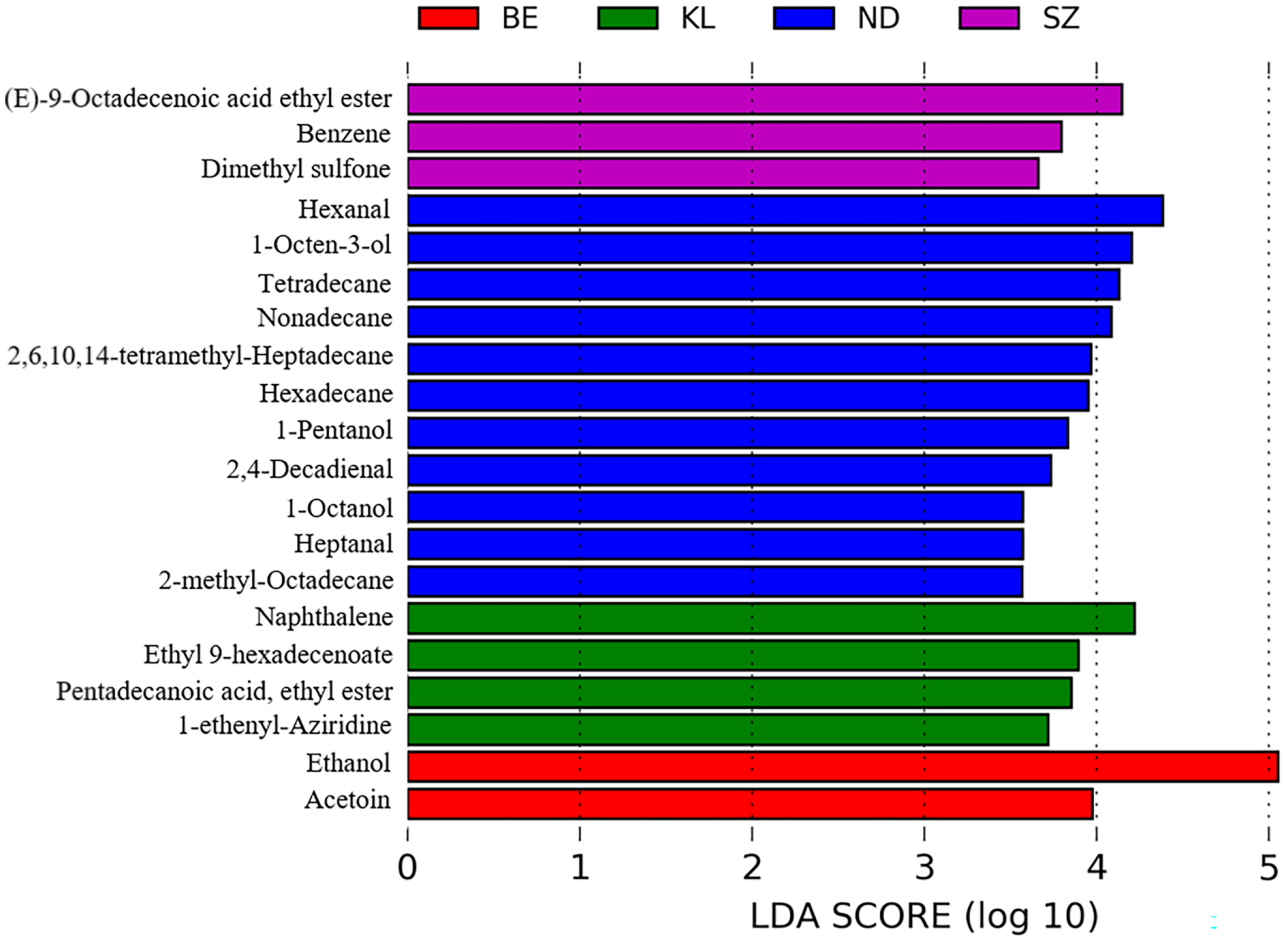
Differential volatile compounds among the four Chinese broiler breeds using LEfSe analysis. BE, Baier yellow chicken; KL, Kangle chicken; ND, Ningdu yellow chicken; SZ, Shengze 901 chicken.

We further compared the flavor composition of chicken meat among the four breeds to identify the flavor compounds associated with each breed. In total, 56 flavor compounds, including 34 flavor compounds that were significantly enriched in Ningdu yellow chickens, five flavor compounds that were significantly enriched in Baier yellow chickens, six flavor compounds that were significantly enriched in Kangle chickens, and 11 flavor compounds that were significantly enriched in Shengze 901 chickens, were identified to be significantly different among the four Chinese broiler breeds using LEfSe analysis (Figure 5; Supplementary Table S3). The flavor compounds enriched in Ningdu yellow chickens mainly belonged to the alcohols (eight compounds: 1-octen-3-ol, (Z)-2-octen-1-ol, 1-pentanol, 1-octanol, 1-hexanol, 1-heptanol, 4-ethylcyclohexanol, and 1-penten-3-ol), hydrocarbons (eight compounds: tetradecane, nonadecane, hexadecane, 2-methyl-octadecane, heptadecane, 2-methyl-eicosane, tridecane, and 3,8-dimethyl-undecane), and aldehydes (six compounds: hexanal, 2,4-decadienal, heptanal, tridecanal, (E, E)-2,4-nonadienal, and propanal). In Shengze 901 chickens, three esters (octadecanoic acid ethyl ester, (E)-9-octadecenoic acid ethyl ester, and 1,1-diethoxy-ethane) and two benzenoids (benzene and benzophenone) were enriched. We then performed a random forest analysis to identify unique flavor biomarkers that could discriminate between the chicken breeds (Supplementary Table S4). A total of 27 flavor compounds differed significantly among the four Chinese chicken breeds as found in the LEfSe analysis (Supplementary Table S5). Specifically, 20 flavor compounds showed significant enrichment in Ningdu yellow chickens, while Baier yellow chickens had two significantly enriched flavor compounds (2-methyl-naphthalene and hexadecanoic acid, methyl ester). Kangle chickens exhibited significant enrichment in four flavor compounds (2,6-lutidine, 3-methyl-tridecane, ethyl 9-hexadecenoate, and pentadecanoic acid, ethyl ester). Additionally, Shengze 901 chickens showed significant enrichment in one flavor compound (benzophenone) (Supplementary Table S5). The flavor compounds enriched in Ningdu yellow chickens were mainly alcohols (six compounds: 1-octen-3-ol, (Z)-2-octen-1-ol, 1-pentanol, 1-octanol, 1-heptanol, and 4-ethylcyclohexanol) and aldehydes (four compounds: (E, E)-2,4-nonadienal, heptanal, tridecanal, and 2,4-decadienal). Jin et al. found that 1-octen-3-ol was the main flavor compound in indigenous high-quality Chinese chickens (27). This result was similar to that of our study where 1-octen-3-ol was significantly enriched in Ningdu yellow chickens. The 1-octen-3-ol, a secondary alcohol with a mushroom-like odor, is considered an important source of the characteristic flavor (28–30). The change in 1-octen-3-ol content is usually used to reflect the degree of racification in meat products (31). Moreover, benzophenone, the only flavor compound significantly enriched in Shengze 901 chickens, is a promising fatty acid synthase inhibitor (32).

### 3.5. Correlations between differentially flavor compounds and phenotypes

**Figure 6:**
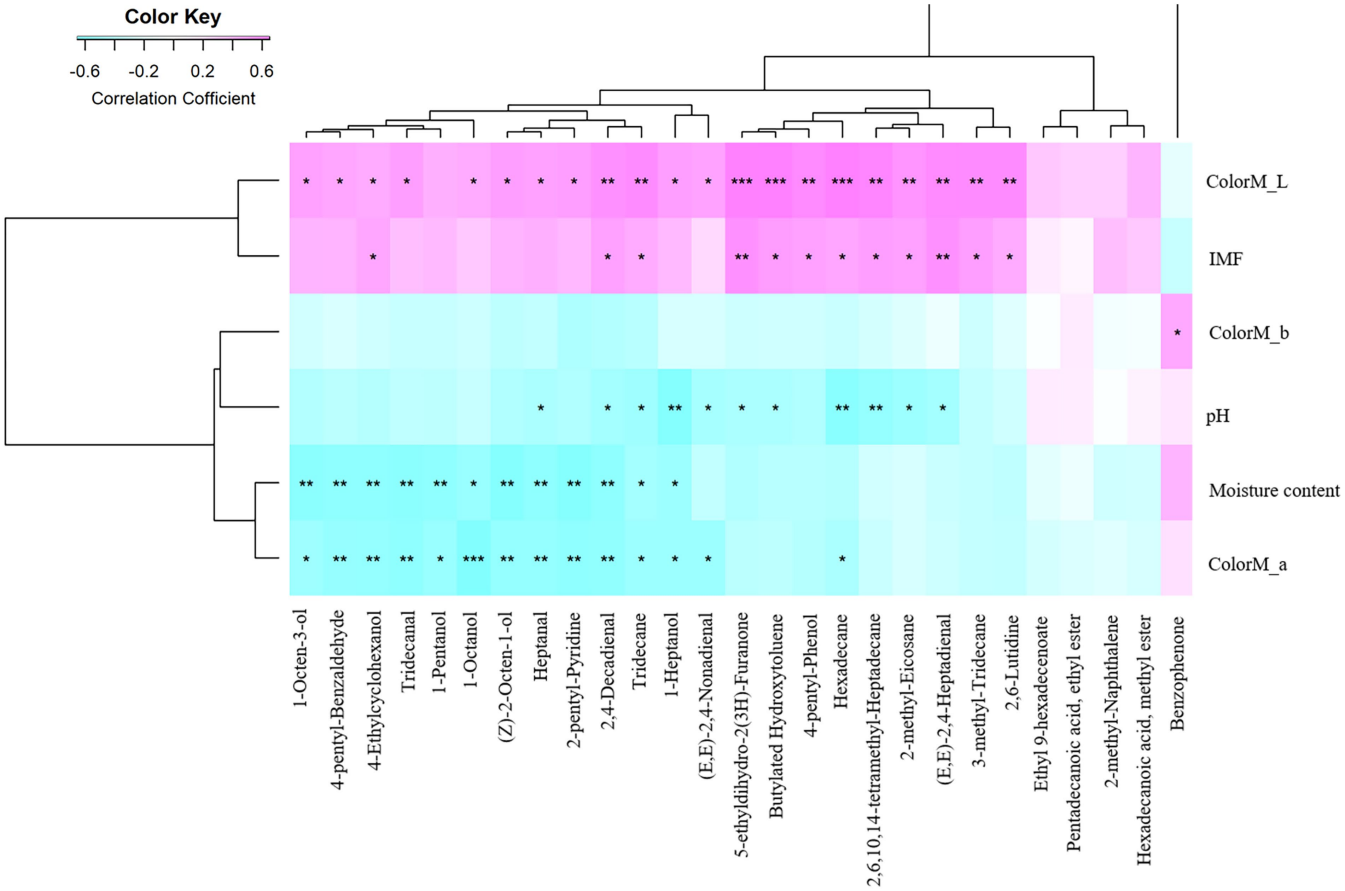
Heat maps showing the relationships between differential volatile compounds and phenotypes of meat quality traits. The *X*-axis represents the volatile compounds. The *Y*-axis indicates the phenotypes of meat quality traits. * *p* < 0.05, ** *p* < 0.01, and *** *p* < 0.001.

Spearman’s correlation analysis was used to determine the relationship between the 27 flavor compounds and six phenotypes of meat quality traits. The results indicated that the two flavor compounds (2-methyl-naphthalene and hexadecanoic acid, methyl ester) enriched in Baier yellow chickens were not significantly correlated with any of the phenotypes studied (Figure 6). Most flavor compounds enriched in Ningdu yellow chickens were significantly correlated with the five phenotypes (except ColorM_b). Moreover, ColorM_L significantly (*p* < 0.001) correlated with 5-ethyldihydro-2(3H)-furanone, hexadecane, and butylated hydroxytoluene. ColorM_a significantly (*p* < 0.001) correlated with 1-octanol. However, ColorM_b significantly (*p* < 0.05) correlated with only benzophenone. Li et al. (8) found that 5-ethyldihydro-2(3H)-furanone, a sugar degradation product formed by Maillard-type reactions, was enriched in *Nuodeng* ham and may contribute to the meaty and sweet flavors of cured hams (33). Hexadecane was found to be a prominent marker for distinguishing between fresh and frozen–thawed chicken thigh meat (34), and between stainless-steel pot chicken soup and the other two types of chicken soup (35). Furthermore, except for ColorM_b, 2,4-decadienal and tridecane were significantly correlated with the other five phenotypes (*p* < 0.05). The most prevalent aldehyde 2,4-decadienal identified in chicken flavor is known to be the primary oxidation product of linoleic acid and is considered a more important odorant for chicken flavor than hexanal because of its much lower odor threshold (36).

## 4. Conclusion

To our knowledge, this is the first study to use GC × GC-TOF-MS to detect the composition and diversity of flavor compounds in chicken meat from four Chinese breeds. A total of 685, 618, 502, and 487 flavor compounds were identified in the Ningdu yellow, Baier yellow, Kangle, and Shengze 901 chickens, respectively. The flavor compounds were separated into eight categories: ketones, hydrocarbons, heterocyclic compounds, aldehydes, esters, alcohols, carboxylic acids, and others. The results also revealed some prominent characteristics of the flavor compounds in Chinese broiler breeds. For example, the hydrocarbons (22.09%) and aldehydes (14.76%) were higher in Ningdu yellow chickens, and alcohols (48.64%) were higher in Baier yellow chickens. The high content of N, N-dimethyl-methylamine in Ningdu yellow, Baier yellow, and Shengze 901 chickens showed the maximum contribution to the overall flavor (ROAV = 439.57, 289.21, and 422.80, respectively). Moreover, 27 flavor compounds that were used as biomarkers for chicken breeds differed significantly among the four Chinese chicken breeds, and most were significantly correlated with meat quality traits. These novel findings provide insights into the unique flavor of chicken meat in indigenous Chinese breeds and are likely to serve as a theoretical reference for the future breeding of desirable chickens.

## Data availability statement

The raw data supporting the conclusions of this article will be made available by the authors, without undue reservation.

## Ethics statement

The animal study was approved by the Ethics Committee of Nanchang Normal University. The study was conducted in accordance with the local legislation and institutional requirements.

## Author contributions

XX: Conceptualization, Data curation, Formal analysis, Funding acquisition, Investigation, Project administration, Supervision, Writing – original draft, Writing – review & editing. JM: Methodology, Writing – review & editing. QH: Methodology, Writing – review & editing. XC: Software, Writing – review & editing. ZW: Software, Writing – review & editing. LL: Formal analysis, Writing – review & editing. JigX: Formal analysis, Writing – review & editing. JinX: Resources, Writing – review & editing. YR: Funding acquisition, Project administration, Resources, Supervision, Writing – review & editing.
