# Supplementary material for: Characteristics and potential biomarkers of flavor compounds in four Chinese indigenous chicken breeds
Source: Front Nutr. 2023 Oct 12;10:1279141. doi: 10.3389/fnut.2023.1279141 (PMC10600453; doi:10.3389/fnut.2023.1279141)
Supplement: Supplementary file 1 [file Data_Sheet_1.docx]

**Supplementary Materials**

**Characteristics and potential biomarkers of flavor compounds in four Chinese indigenous chicken breeds**

Xinwei Xiong^1,†,^*, Jinge Ma^1,†^, Qin He^1^, Xiaolian Chen^2^, Zhangfeng Wang^1^, Longyun Li^1^, Jiguo Xu^1^, Jinfang Xie^2^, Yousheng Rao^1^*

*^1^Key Laboratory for Genetic Improvement of Indigenous Chicken Breeds of Jiangxi Province, Nanchang Normal University, Nanchang, 330032, China.*

*^2^Institute of Animal Husbandry and Veterinary Medicine, Jiangxi Academy of Agricultural Sciences, Nanchang, 330200, China.*

†These authors contributed equally to this work.

*Corresponding author: Xinwei Xiong and Yousheng Rao

Key Laboratory for Genetic Improvement of Indigenous Chicken Breeds of Jiangxi Province, Nanchang Normal University, Nanchang, 330032, China.

Phone: 0086-791- 83812115

Fax: 0086-791- 83812115

E-mail: [xinweixiong@hotmail.com](mailto:xinweixiong@hotmail.com) and rys8323571@aliyun.com


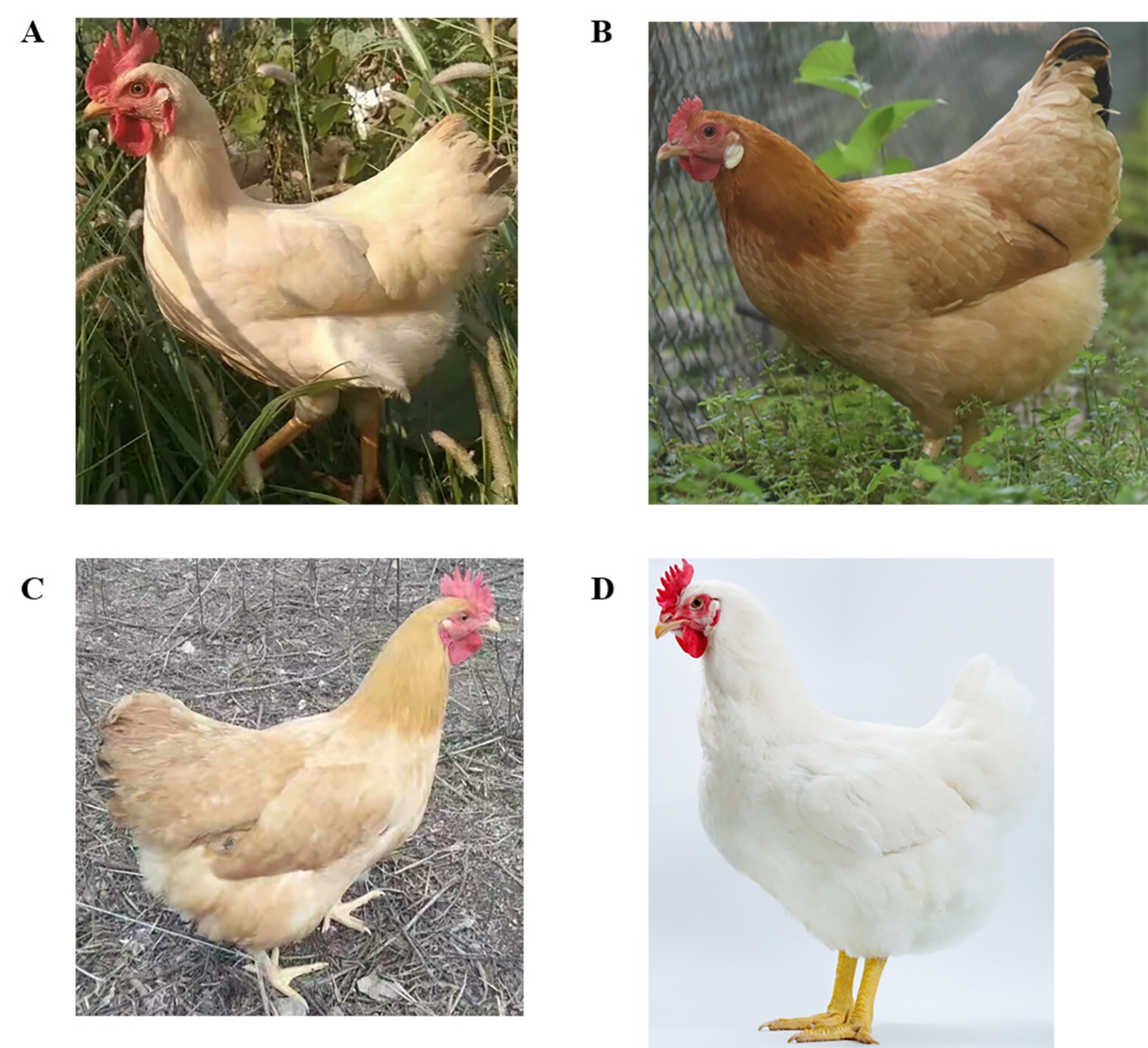


**Supplementary Fig. S1 The photograph of four Chinese chicken breeds.** (A) Ningdu yellow chicken. (B) Baier yellow chicken. (C) Kangle chicken. (D) Shengze 901 chicken.


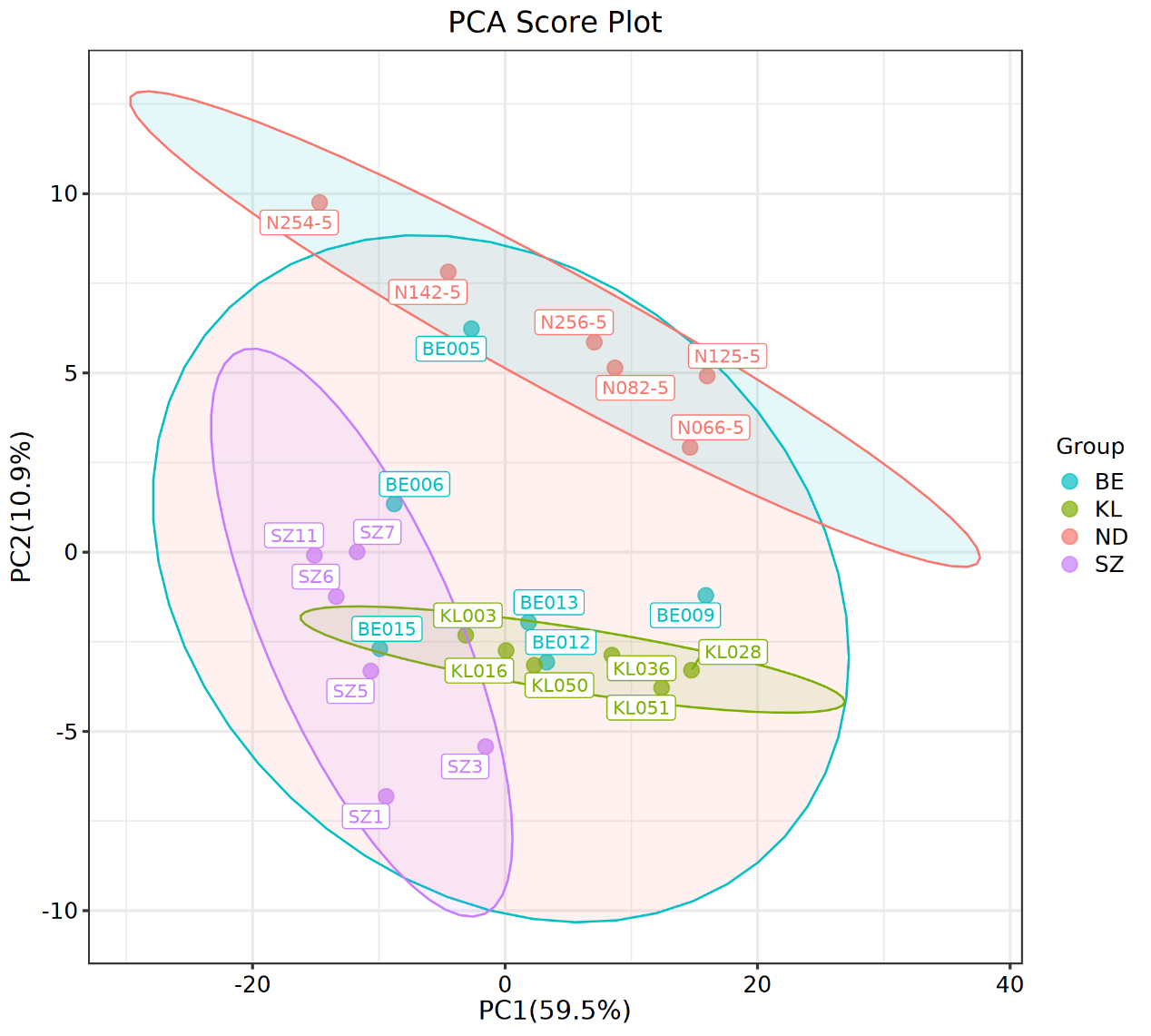


**Supplementary Fig. S2 PCA plot in samples from four Chinese chicken breeds.** BE: Baier yellow chicken, KL: Kangle chicken, ND: Ningdu yellow chicken, SZ: Shengze 901 chicken.


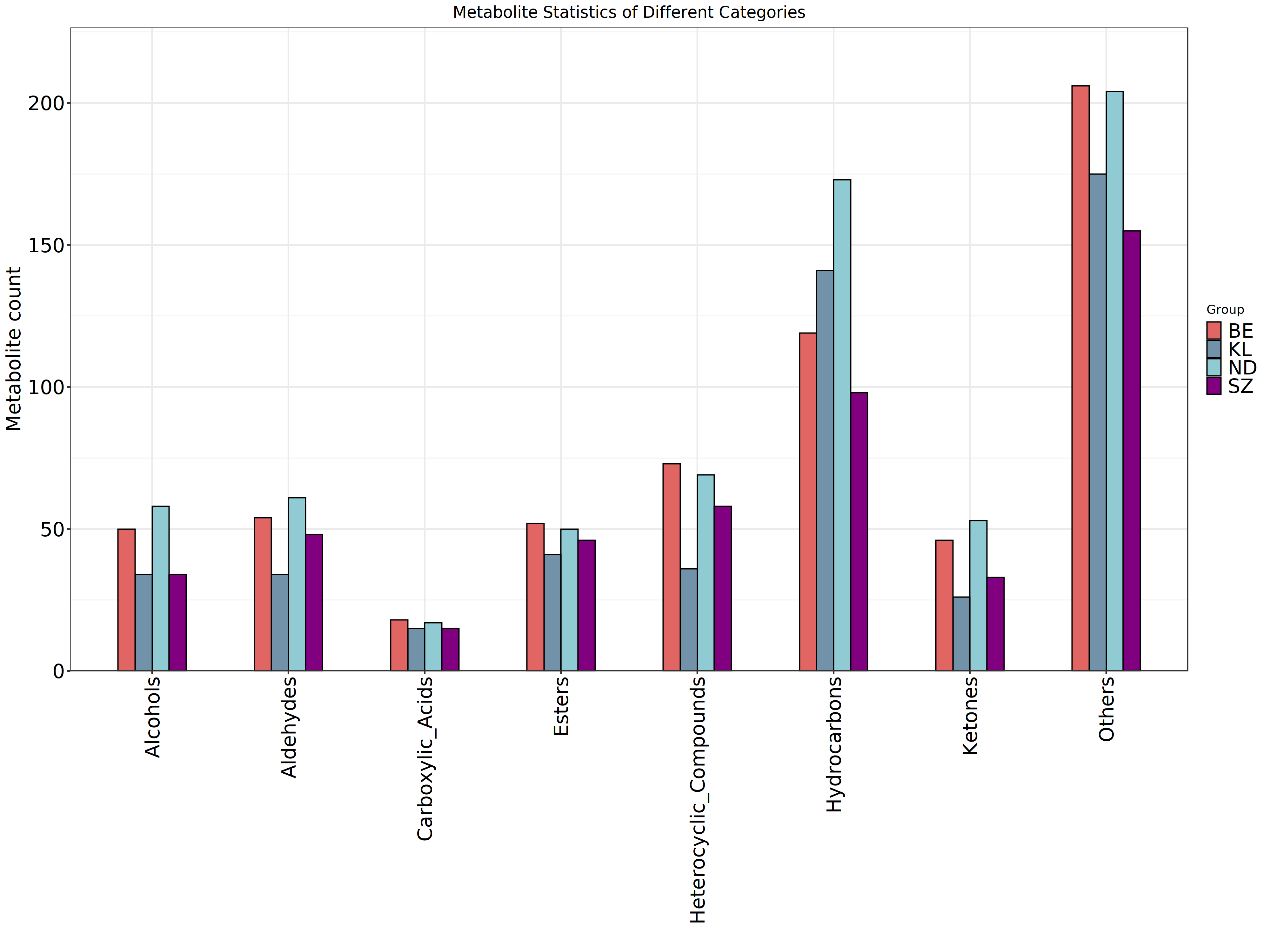


**Supplementary Fig. S3 The flavor compounds statistics of different categories.** BE: Baier yellow chicken, KL: Kangle chicken, ND: Ningdu yellow chicken, SZ: Shengze 901 chicken.


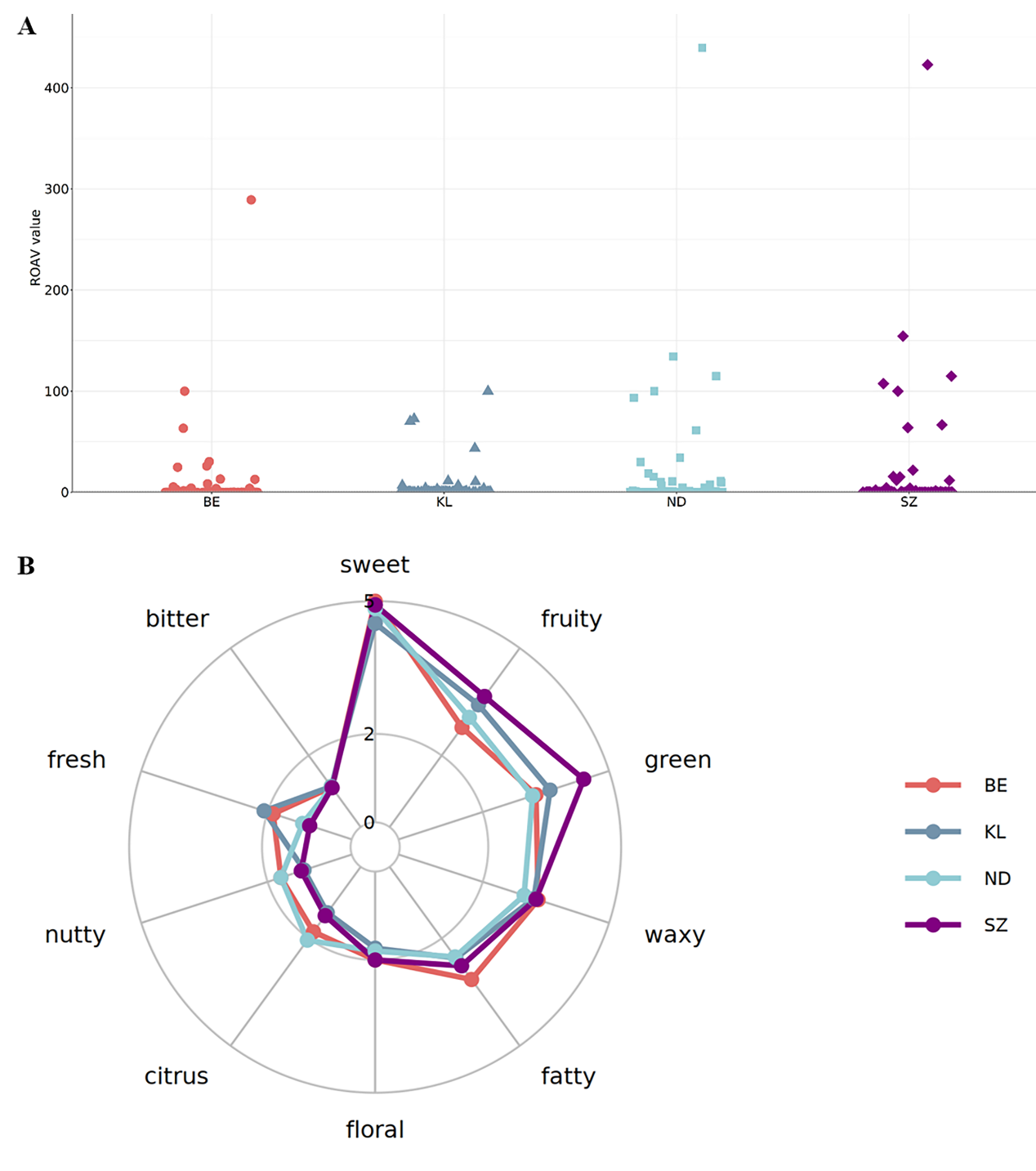


**Supplementary Fig. S4 Flavor compounds threshold and sensory flavor characteristics analyses.** (A) ROAV analysis, (B) FlavorDB analysis. BE: Baier yellow chicken, KL: Kangle chicken, ND: Ningdu yellow chicken, SZ: Shengze 901 chicken.


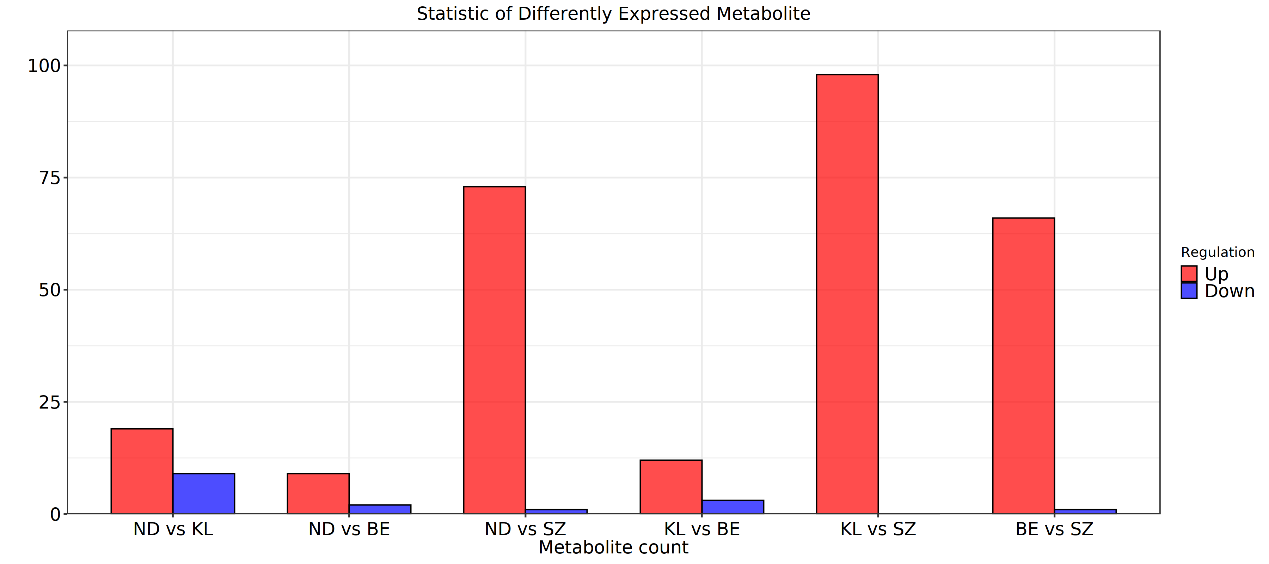


**Supplementary Fig. S5 The flavor compounds with statistical significance (P < 0.05, and VIP > 1) between any two of the four Chinese broiler breeds.** The red representing upregulated differential flavor compounds, blue representing downregulated differential flavor compounds. BE: Baier yellow chicken, KL: Kangle chicken, ND: Ningdu yellow chicken, SZ: Shengze 901 chicken.

**Supplementary Table S1. Ingredient composition of diet for all chickens.**

| Ingredient, % | diet |
| --- | --- |
| Maize | 62 |
| Soybean meal | 21 |
| Wheat bran | 5 |
| Rice bran | 2 |
| Calcium hydrogen phosphate | 1.7 |
| Stone powder | 7 |
| 5% pre powder | 1 |
| Salt | 0.3 |
| Total | 100 |
| Composition |  |
| Digestible energy (MJ/kg) | 11.42 |
| Crude protein (%) | 15.58 |
| Calcium (%) | 3.01 |
| Phosphorus (%) | 0.64 |
| Lysine (%) | 0.71 |
| Methionine (%) | 0.23 |

**Supplementary Table S2 ROAV analysis of flavor compounds in chicken meat from four Chinese breeds.**

| **Name** | **Class** | **CAS** | **Formula** | **ND_ROAV** | **KL_ROAV** | **BE_ROAV** | **SZ_ROAV** |
| --- | --- | --- | --- | --- | --- | --- | --- |
| 2,3-Butanedione | Ketones | 431-03-8 | C4H6O2 | 100 | 100 | 100 | 100 |
| Benzene, 1,2,4-trimethyl- | Benzenoids | 95-63-6 | C9H12 | 0.016934 | 0.032304 | 0.005729 | 0.016699 |
| Propane, 1,2-dichloro- | Organohalogen compounds | 78-87-5 | C3H6Cl2 | 0.000633 | 0.000285 | 0.000132 | 0.000385 |
| 1,3-Dioxolane | Heterocyclic_Compounds | 646-06-0 | C3H6O2 | 6.05E-06 | 4.41E-06 | 2.05E-06 | 0.000104 |
| Benzene, 1,4-dichloro- | Benzenoids | 106-46-7 | C6H4Cl2 | 0.00084 | 0.000612 | 0.000284 | 0.000829 |
| 1-Butanol | Alcohols | 71-36-3 | C4H10O | 0.030788 | 0.022442 | 0.011312 | 0.032615 |
| 1-Butene | Hydrocarbons | 106-98-9 | C4H8 | 0.000281 | 0.000205 | 9.5E-05 | 0.000292 |
| 1-Decene | Hydrocarbons | 872-05-9 | C10H20 | 0.000154 | 1.18E-05 | 8.05E-06 | 0.001069 |
| 1-Hexanol | Alcohols | 111-27-3 | C6H14O | 9.665982 | 0.172901 | 1.461771 | 0.827964 |
| 2-Propanol, 1-methoxy- | Alcohols | 107-98-2 | C4H10O2 | 1.64E-05 | 1.13E-05 | 6.78E-06 | 1.19E-05 |
| 1-Pentanol | Alcohols | 71-41-0 | C5H12O | 9.944948 | 0.086822 | 1.286232 | 0.934521 |
| 1-Octanol | Alcohols | 111-87-5 | C8H18O | 34.22904 | 0.532344 | 3.049013 | 1.856723 |
| 1-Octene | Hydrocarbons | 111-66-0 | C8H16 | 0.101601 | 0.07406 | 0.036212 | 0.100195 |
| 2-Heptanone | Ketones | 110-43-0 | C7H14O | 10.70525 | 0.306547 | 3.862142 | 0.136976 |
| 2-Hexanone | Ketones | 591-78-6 | C6H12O | 0.004233 | 0.003409 | 0.001432 | 0.004175 |
| Pyridine, 2-methyl- | Heterocyclic_Compounds | 109-06-8 | C6H7N | 0.08893 | 0.028485 | 0.014009 | 0.038536 |
| Naphthalene, 2-methyl- | Benzenoids | 91-57-6 | C11H10 | 0.824524 | 0.243858 | 8.402813 | 0.576054 |
| Pentane, 2-methyl- | Hydrocarbons | 107-83-5 | C6H14 | 0.000239 | 0.000184 | 8.07E-05 | 0.000235 |
| Ethanol, 2-methoxy- | Ethers | 109-86-4 | C3H8O2 | 0.000566 | 0.001259 | 2.99E-06 | 0.000124 |
| 2-Pentanone | Ketones | 107-87-9 | C5H10O | 0.025546 | 0.004453 | 0.011676 | 0.040022 |
| Ethanol, 2-ethoxy- | Ethers | 110-80-5 | C4H10O2 | 0.000339 | 0.00026 | 0.000121 | 0.000334 |
| Citral | Lipids and lipid-like molecules | 5392-40-5 | C10H16O | 4.500102 | 3.085842 | 1.54635 | 4.174787 |
| 2-Butanone, 3-methyl- | Ketones | 563-80-4 | C5H10O | 0.001115 | 0.000145 | 6.75E-05 | 0.000196 |
| 2-Pentanol, 4-methyl- | Alcohols | 108-11-2 | C6H14O | 0.000303 | 0.000227 | 0.000103 | 0.000299 |
| 3-Heptanone, 5-methyl- | Ketones | 541-85-5 | C8H16O | 0.000855 | 1.41E-05 | 5.9E-06 | 8.95E-05 |
| a-Methylstyrene | Benzenoids | 98-83-9 | C9H10 | 0.00508 | 0.008011 | 0.004403 | 0.00501 |
| Benzene | Benzenoids | 71-43-2 | C6H6 | 0.015622 | 0.015312 | 0.01307 | 0.153937 |
| Aniline | Benzenoids | 62-53-3 | C6H7N | 0.008467 | 0.006172 | 0.002865 | 0.011127 |
| Phenol | Benzenoids | 108-95-2 | C6H6O | 1.370531 | 0.689862 | 0.343034 | 1.443229 |
| Benzaldehyde | Benzenoids | 100-52-7 | C7H6O | 93.33493 | 43.53327 | 24.79092 | 66.61562 |
| Acetophenone | Ketones | 98-86-2 | C8H8O | 18.62977 | 10.40412 | 4.108375 | 11.79904 |
| Styrene | Benzenoids | 100-42-5 | C8H8 | 0.756251 | 0.245971 | 0.11306 | 0.332491 |
| Pyridine | Heterocyclic_Compounds | 110-86-1 | C5H5N | 0.291821 | 0.081185 | 0.108889 | 0.235929 |
| 1-Propanol | Alcohols | 71-23-8 | C3H8O | 2.69E-08 | 2.2E-08 | 1.11E-07 | 1.22E-06 |
| Propanal | Aldehydes | 123-38-6 | C3H6O | 4.471614 | 0.078473 | 0.692342 | 1.580204 |
| Acetone | Ketones | 67-64-1 | C3H6O | 0.112483 | 0.04715 | 0.039335 | 0.195024 |
| Propene | Hydrocarbons | 115-07-1 | C3H6 | 1.01E-05 | 7.33E-06 | 3.4E-06 | 1.04E-05 |
| 2-Propenenitrile | Organonitrogen compounds | 107-13-1 | C3H3N | 6.35E-05 | 4.63E-05 | 2.2E-05 | 6.26E-05 |
| 2-Propenal | Organic oxygen compounds | 107-02-8 | C3H4O | 0.503286 | 0.195141 | 0.137109 | 0.555598 |
| 2-Propenoic acid, ethyl ester | Esters | 140-88-5 | C5H8O2 | 15.3941 | 11.22124 | 5.341464 | 15.18104 |
| Ethylenimine | Heterocyclic_Compounds | 151-56-4 | C2H5N | 0.000143 | 0.000104 | 5.01E-05 | 0.119076 |
| Butanal | Aldehydes | 123-72-8 | C4H8O | 6.408201 | 0.246867 | 0.151555 | 0.333983 |
| 1,4-Dioxane | Heterocyclic_Compounds | 123-91-1 | C4H8O2 | 0.000127 | 9.26E-05 | 4.47E-05 | 0.000125 |
| Ethanol, 2-(2-ethoxyethoxy)- | Ethers | 111-90-0 | C6H14O3 | 0.000962 | 0.000437 | 0.000189 | 0.00222 |
| Disulfide, dimethyl | Organosulfur compounds | 624-92-0 | C2H6S2 | 1.48575 | 0.446921 | 0.367308 | 0.345816 |
| Dimethyl sulfide | Ethers | 75-18-3 | C2H6S | 0.846675 | 0.617168 | 0.571262 | 107.4737 |
| Dimethyl ether | Ethers | 115-10-6 | C2H6O | 5.29E-06 | 0.000514 | 1.06E-05 | 0.002879 |
| Ethanamine, N-ethyl- | Organonitrogen compounds | 109-89-7 | C4H11N | 0.306822 | 0.098256 | 0.010417 | 0.031726 |
| 4-Heptanone, 2,6-dimethyl- | Ketones | 108-83-8 | C9H18O | 0.000113 | 8.23E-05 | 3.82E-05 | 0.000111 |
| 1-Butanamine, N-butyl- | Organic nitrogen compounds | 111-92-2 | C8H19N | 0.007443 | 0.023708 | 0.007013 | 0.004332 |
| Heptane | Hydrocarbons | 142-82-5 | C7H16 | 0.003802 | 0.000181 | 0.000563 | 0.000244 |
| Cyclohexanol | Alcohols | 108-93-0 | C6H12O | 0.001752 | 0.00365 | 0.000593 | 0.001727 |
| Cyclohexanone | Ketones | 108-94-1 | C6H10O | 0.001954 | 0.064771 | 0.000661 | 0.001927 |
| Caprolactam | Heterocyclic_Compounds | 105-60-2 | C6H11NO | 0.001584 | 0.001495 | 0.001753 | 0.001541 |
| n-Hexane | Hydrocarbons | 110-54-3 | C6H14 | 0.000686 | 0.003259 | 0.002377 | 0.00074 |
| Toluene | Benzenoids | 108-88-3 | C7H8 | 0.149747 | 0.041874 | 0.054988 | 0.129718 |
| Hydrazine, methyl- | Organonitrogen compounds | 60-34-4 | CH6N2 | 0.00038 | 0.097269 | 0.02983 | 0.060179 |
| 2-Butanone | Ketones | 78-93-3 | C4H8O | 0.265158 | 0.12891 | 0.100776 | 0.45565 |
| Methyl vinyl ketone | Ketones | 78-94-4 | C4H6O | 0.000584 | 0.000426 | 0.000201 | 0.000576 |
| Methane, isocyanato- | Organonitrogen compounds | 624-83-9 | C2H3NO | 4.75E-05 | 3.46E-05 | 1.61E-05 | 4.78E-05 |
| Methanethiol |  | 74-93-1 | CH4S | 0.050493 | 0.030916 | 0.027546 | 0.064517 |
| Formic acid | Carboxylic_Acids | 64-18-6 | CH2O2 | 0.007406 | 0.000505 | 6.72E-05 | 0.001328 |
| Methyl formate | Esters | 107-31-3 | C2H4O2 | 1.52E-06 | 2.01E-05 | 5.13E-07 | 1.5E-06 |
| Ethyl formate | Esters | 109-94-4 | C3H6O2 | 0.00121 | 0.000908 | 0.000507 | 0.000319 |
| Benzene, (1-methylethyl)- | Benzenoids | 98-82-8 | C9H12 | 0.022155 | 0.009258 | 0.004297 | 0.012524 |
| Biphenyl | Benzenoids | 92-52-4 | C12H10 | 0.880684 | 0.470934 | 0.692369 | 0.758205 |
| Dibutyl phthalate | Esters | 84-74-2 | C16H22O4 | 0.004475 | 0.033609 | 0.036025 | 0.07964 |
| Diethyl Phthalate | Esters | 84-66-2 | C12H14O4 | 0.002822 | 0.002057 | 0.000956 | 0.002783 |
| Trichloromethane | Organohalogen compounds | 67-66-3 | CHCl3 | 0.001754 | 0.02279 | 0.016558 | 0.00609 |
| Naphthalene | Benzenoids | 91-20-3 | C10H8 | 61.38574 | 70.28986 | 30.31961 | 63.93276 |
| Limonene | Lipids and lipid-like molecules | 138-86-3 | C10H16 | 0.05732 | 0.042813 | 0.019122 | 0.055664 |
| Nonane | Hydrocarbons | 111-84-2 | C9H20 | 7.9E-05 | 0.000234 | 0.000136 | 0.000537 |
| Methylamine, N,N-dimethyl- | Organonitrogen compounds | 75-50-3 | C3H9N | 439.5717 | 3.70301 | 289.2128 | 422.7993 |
| 2-Propanol, 2-methyl- | Alcohols | 75-65-0 | C4H10O | 0.000289 | 0.000183 | 6.58E-05 | 0.000144 |
| Tetrahydrofuran | Heterocyclic_Compounds | 109-99-9 | C4H8O | 0.062921 | 0.010704 | 0.001218 | 0.001091 |
| Pentanal | Aldehydes | 110-62-3 | C5H10O | 134.2673 | 1.098938 | 26.08386 | 15.62224 |
| 2-Propen-1-ol | Alcohols | 107-18-6 | C3H6O | 0.000199 | 0.000145 | 7.07E-05 | 0.000197 |
| Benzene, nitro- | Benzenoids | 98-95-3 | C6H5NO2 | 0.254003 | 0.192294 | 0.085942 | 0.250487 |
| Octane | Hydrocarbons | 111-65-9 | C8H18 | 0.004835 | 0.000423 | 0.000397 | 0.000584 |
| Ethylbenzene | Benzenoids | 100-41-4 | C8H10 | 1.49E-05 | 5.53E-05 | 9.99E-06 | 1.1E-05 |
| Ethanol | Alcohols | 64-17-5 | C2H6O | 11.29446 | 6.855451 | 12.80204 | 21.89247 |
| Ethyl ether | Ethers | 60-29-7 | C4H10O | 0.001408 | 0.001463 | 0.001671 | 0.001555 |
| Acetaldehyde | Aldehydes | 75-07-0 | C2H4O | 114.8377 | 72.67932 | 63.311 | 154.4378 |
| Acetic acid | Carboxylic_Acids | 64-19-7 | C2H4O2 | 30.03694 | 6.733973 | 13.07233 | 114.8472 |
| n-Propyl acetate | Esters | 109-60-4 | C5H10O2 | 0.002117 | 0.001543 | 0.000721 | 0.002104 |
| Acetic acid, butyl ester | Esters | 123-86-4 | C6H12O2 | 0.781546 | 0.569694 | 0.265354 | 0.77073 |
| Acetic anhydride | Carboxylic_Acids | 108-24-7 | C4H6O3 | 0.000847 | 0.008776 | 0.000286 | 0.00796 |
| Acetic acid, pentyl ester | Esters | 628-63-7 | C7H14O2 | 0.014514 | 0.01058 | 0.004911 | 0.014409 |
| Acetic acid ethenyl ester | Esters | 108-05-4 | C4H6O2 | 0.063251 | 0.000617 | 0.047033 | 0.416661 |
| Ethyl Acetate | Esters | 141-78-6 | C4H8O2 | 0.042261 | 0.062052 | 0.082707 | 0.628962 |
| Isopropyl acetate | Esters | 108-21-4 | C5H10O2 | 0.000635 | 0.000463 | 0.000229 | 0.000632 |
| Isopropyl Alcohol | Alcohols | 67-63-0 | C3H8O | 0.090412 | 0.161999 | 0.003991 | 0.086582 |
| 1-Propanol, 2-methyl- | Alcohols | 78-83-1 | C4H10O | 0.01016 | 0.007406 | 0.003527 | 0.022912 |
| Propanal, 2-methyl- | Aldehydes | 78-84-2 | C4H8O | 7.681902 | 4.148593 | 3.706986 | 11.67718 |
| 1-Butanol, 3-methyl- | Alcohols | 123-51-3 | C5H12O | 0.060237 | 0.043823 | 0.031389 | 4.421752 |
| 2-Butenal | Organic oxygen compounds | 4170-30-3 | C4H6O | 0.005099 | 0.003703 | 0.00687 | 0.005013 |
| 2-Propanamine |  | 75-31-0 | C3H9N | 0.076265 | 0.30029 | 0.002613 | 2.200915 |
| Phosgene |  | 75-44-5 | CCl2O | 0.000847 | 0.031635 | 0.000286 | 0.000835 |

**Supplementary Table S3 Flavor compounds showing significantly different among the four Chinese broiler breeds using LEfSe analysis.**

| **Name** | **Class** | **Group** | **LDA value** | **P value** |
| --- | --- | --- | --- | --- |
| Ethanol | Alcohols | BE | 5.05 | 2.31E-03 |
| Hexanal | Aldehydes | ND | 4.39 | 3.01E-03 |
| Naphthalene | Benzenoids | KL | 4.22 | 8.10E-03 |
| 1-Octen-3-ol | Alcohols | ND | 4.20 | 9.01E-04 |
| (E)-9-Octadecenoic acid ethyl ester | Esters | SZ | 4.15 | 9.18E-04 |
| Tetradecane | Hydrocarbons | ND | 4.13 | 5.98E-03 |
| Nonadecane | Hydrocarbons | ND | 4.09 | 4.75E-03 |
| Acetoin | Organic oxygen compounds | BE | 3.97 | 4.71E-03 |
| Heptadecane, 2,6,10,14-tetramethyl- | Lipids and lipid-like molecules | ND | 3.97 | 8.28E-04 |
| Hexadecane | Hydrocarbons | ND | 3.95 | 3.45E-03 |
| Ethyl 9-hexadecenoate | Esters | KL | 3.89 | 9.72E-04 |
| Pentadecanoic acid, ethyl ester | Esters | KL | 3.85 | 2.03E-03 |
| 1-Pentanol | Alcohols | ND | 3.83 | 5.61E-04 |
| Benzene | Benzenoids | SZ | 3.80 | 1.24E-03 |
| 2,4-Decadienal | Aldehydes | ND | 3.74 | 3.64E-03 |
| Aziridine, 1-ethenyl- | Heterocyclic_Compounds | KL | 3.72 | 8.48E-03 |
| Dimethyl sulfone | Organosulfur compounds | SZ | 3.66 | 1.12E-03 |
| 1-Octanol | Alcohols | ND | 3.57 | 4.19E-04 |
| Heptanal | Aldehydes | ND | 3.57 | 2.32E-03 |
| Octadecane, 2-methyl- | Hydrocarbons | ND | 3.57 | 7.71E-03 |
| Tridecanal | Aldehydes | ND | 3.57 | 1.50E-03 |
| 2,6-Lutidine | Heterocyclic_Compounds | KL | 3.55 | 9.84E-03 |
| 2(3H)-Furanone, 5-ethyldihydro- | Heterocyclic_Compounds | ND | 3.47 | 3.64E-03 |
| 7,9-Di-tert-butyl-1-oxaspiro(4,5)deca-6,9-diene-2,8-dione | Heterocyclic_Compounds | SZ | 3.47 | 2.98E-03 |
| 2-Octen-1-ol, (Z)- | Alcohols | ND | 3.47 | 1.92E-03 |
| 1-Hexanol | Alcohols | ND | 3.45 | 4.40E-04 |
| Benzaldehyde, 4-pentyl- | Benzenoids | ND | 3.44 | 1.73E-03 |
| Ethanone, 1,1'-(1,4-phenylene)bis- | Ketones | ND | 3.44 | 2.77E-03 |
| Heptadecane | Hydrocarbons | ND | 3.43 | 3.91E-03 |
| Hexadecanoic acid, methyl ester | Esters | BE | 3.42 | 1.02E-03 |
| Eicosane, 2-methyl- | Hydrocarbons | ND | 3.38 | 1.85E-03 |
| Octadecanoic acid, ethyl ester | Esters | SZ | 3.38 | 2.10E-03 |
| 1-Heptanol | Alcohols | ND | 3.33 | 1.14E-03 |
| 4-Ethylcyclohexanol | Alcohols | ND | 3.33 | 2.28E-03 |
| Pyridine, 2-pentyl- | Heterocyclic_Compounds | ND | 3.32 | 6.30E-03 |
| Butylated Hydroxytoluene | Benzenoids | ND | 3.28 | 1.59E-03 |
| Tridecane | Hydrocarbons | ND | 3.24 | 2.59E-03 |
| Pyridine | Heterocyclic_Compounds | BE | 3.23 | 6.88E-03 |
| Butanal, 3-methyl- | Aldehydes | SZ | 3.22 | 3.83E-03 |
| 2,4-Nonadienal, (E,E)- | Aldehydes | ND | 3.20 | 2.07E-03 |
| Ethanone, 1-(3,4-dimethylphenyl)- |  | ND | 3.20 | 4.36E-03 |
| 2-Tridecanone | Ketones | SZ | 3.18 | 7.41E-03 |
| 2,4-Heptadienal, (E,E)- |  | ND | 3.15 | 4.29E-03 |
| Benzophenone | Benzenoids | SZ | 3.12 | 2.62E-03 |
| Tetradecanoic acid | Lipids and lipid-like molecules | SZ | 3.07 | 4.06E-03 |
| Ethane, 1,1-diethoxy- | Ethers | SZ | 3.07 | 3.04E-03 |
| Naphthalene, 2-methyl- | Benzenoids | BE | 3.04 | 4.37E-03 |
| Tridecane, 3-methyl- | Hydrocarbons | KL | 3.02 | 1.74E-03 |
| 1-Methylimidazole-5-carboxaldehyde | Heterocyclic_Compounds | ND | 3.01 | 2.92E-03 |
| 1-Penten-3-ol | Alcohols | ND | 3.01 | 6.71E-03 |
| Nonanoic acid | Lipids and lipid-like molecules | SZ | 3.00 | 5.91E-03 |
| Propanal | Aldehydes | ND | 2.99 | 6.83E-03 |
| Formic acid, dodecyl ester | Esters | ND | 2.93 | 8.30E-03 |
| Nonanoic acid, ethyl ester | Esters | ND | 2.92 | 1.07E-03 |
| Phenol, 4-pentyl- | Benzenoids | ND | 2.91 | 7.69E-03 |
| Undecane, 3,8-dimethyl- | Hydrocarbons | ND | 2.90 | 8.62E-03 |

**Supplementary Table S4 Flavor compounds showing significantly different among the four Chinese broiler breeds using random forest analysis.**

| Name | Class | MeanDecreaseAccuracy | MeanDecreaseGini |
| --- | --- | --- | --- |
| 1-Heptanol | Alcohols | 7.83 | 0.57 |
| Benzaldehyde, 4-pentyl- | Benzenoids | 6.50 | 0.39 |
| 2,4-Decadienal | Aldehydes | 6.29 | 0.48 |
| Eicosane, 2-methyl- | Hydrocarbons | 6.17 | 0.31 |
| 4-Ethylcyclohexanol | Alcohols | 5.56 | 0.33 |
| 2(3H)-Furanone, 5-ethyldihydro- | Heterocyclic_Compounds | 5.49 | 0.35 |
| 2-Octen-1-ol, (Z)- | Alcohols | 5.48 | 0.29 |
| Tridecane, 3-methyl- | Hydrocarbons | 5.05 | 0.23 |
| Naphthalene, 2-methyl- | Benzenoids | 4.90 | 0.26 |
| Ethyl 9-hexadecenoate | Esters | 4.72 | 0.28 |
| Pyridine, 2-pentyl- | Heterocyclic_Compounds | 4.69 | 0.24 |
| 1-Octanol | Alcohols | 4.45 | 0.33 |
| Butylated Hydroxytoluene | Benzenoids | 4.44 | 0.23 |
| 1-Butanamine, N-butyl- | Organic nitrogen compounds | 4.29 | 0.25 |
| (E)-9-Octadecenoic acid ethyl ester | Esters | 4.22 | 0.29 |
| Nonanoic acid, ethyl ester | Esters | 4.21 | 0.31 |
| 7,9-Di-tert-butyl-1-oxaspiro(4,5)deca-6,9-diene-2,8-dione | Heterocyclic_Compounds | 3.99 | 0.12 |
| Tridecanal | Aldehydes | 3.93 | 0.32 |
| 1-Octen-3-ol | Alcohols | 3.66 | 0.25 |
| Hexadecanoic acid, methyl ester | Esters | 3.65 | 0.14 |
| Decane, 2,4-dimethyl- | Hydrocarbons | 3.61 | 0.21 |
| 2-Naphthyl methyl ketone |  | 3.51 | 0.13 |
| 1-Pentanol | Alcohols | 3.49 | 0.26 |
| Heptadecane, 2,6,10,14-tetramethyl- | Lipids and lipid-like molecules | 3.45 | 0.16 |
| Hexadecane | Hydrocarbons | 3.39 | 0.16 |
| p-Xylene | Benzenoids | 3.29 | 0.14 |
| Niacinamide | Heterocyclic_Compounds | 3.15 | 0.12 |
| 2,4-Nonadienal, (E,E)- | Aldehydes | 3.08 | 0.20 |
| Benzophenone | Benzenoids | 3.06 | 0.13 |
| Tridecane | Hydrocarbons | 3.05 | 0.15 |
| Phenol, 4-pentyl- | Benzenoids | 2.98 | 0.14 |
| 2,6-Lutidine | Heterocyclic_Compounds | 2.97 | 0.11 |
| Pentadecanoic acid, ethyl ester | Esters | 2.96 | 0.10 |
| Ethanone, 1-(2-pyridinyl)- | Ketones | 2.96 | 0.12 |
| Decanoic acid, ethyl ester | Esters | 2.89 | 0.12 |
| Octane, 2,4,6-trimethyl- | Hydrocarbons | 2.89 | 0.13 |
| Heptanal | Aldehydes | 2.83 | 0.15 |
| Tridecane, 6-methyl- | Hydrocarbons | 2.80 | 0.11 |
| 2-Heptenal, (Z)- | Aldehydes | 2.75 | 0.14 |
| 3,5-Octadien-2-one, (E,E)- | Ketones | 2.68 | 0.14 |
| 1-Tetradecene | Hydrocarbons | 2.61 | 0.15 |
| Pentadecanal- |  | 2.55 | 0.12 |
| 2,4-Heptadienal, (E,E)- |  | 2.53 | 0.11 |
| Tetradecanoic acid, ethyl ester | Esters | 2.52 | 0.13 |

**Supplementary Table S5 The overlap flavor compounds through LEfSe and random forest analyses.**

| **Name** | **Class** | **MeanDecreaseAccuracy** | **MeanDecreaseGini** | **LDA value** | **Group** |
| --- | --- | --- | --- | --- | --- |
| 1-Heptanol | Alcohols | 7.83 | 0.57 | 3.33 | ND |
| Benzaldehyde, 4-pentyl- | Benzenoids | 6.50 | 0.39 | 3.44 | ND |
| 2,4-Decadienal | Aldehydes | 6.29 | 0.48 | 3.74 | ND |
| Eicosane, 2-methyl- | Hydrocarbons | 6.17 | 0.31 | 3.38 | ND |
| 4-Ethylcyclohexanol | Alcohols | 5.56 | 0.33 | 3.33 | ND |
| 2(3H)-Furanone, 5-ethyldihydro- | Heterocyclic_Compounds | 5.49 | 0.35 | 3.47 | ND |
| 2-Octen-1-ol, (Z)- | Alcohols | 5.48 | 0.29 | 3.47 | ND |
| Tridecane, 3-methyl- | Hydrocarbons | 5.05 | 0.23 | 3.02 | KL |
| Naphthalene, 2-methyl- | Benzenoids | 4.90 | 0.26 | 3.04 | BE |
| Ethyl 9-hexadecenoate | Esters | 4.72 | 0.28 | 3.89 | KL |
| Pyridine, 2-pentyl- | Heterocyclic_Compounds | 4.69 | 0.24 | 3.32 | ND |
| 1-Octanol | Alcohols | 4.45 | 0.33 | 3.57 | ND |
| Butylated Hydroxytoluene | Benzenoids | 4.44 | 0.23 | 3.28 | ND |
| Tridecanal | Aldehydes | 3.93 | 0.32 | 3.57 | ND |
| 1-Octen-3-ol | Alcohols | 3.66 | 0.25 | 4.20 | ND |
| Hexadecanoic acid, methyl ester | Esters | 3.65 | 0.14 | 3.42 | BE |
| 1-Pentanol | Alcohols | 3.49 | 0.26 | 3.83 | ND |
| Heptadecane, 2,6,10,14-tetramethyl- | Lipids and lipid-like molecules | 3.45 | 0.16 | 3.97 | ND |
| Hexadecane | Hydrocarbons | 3.39 | 0.16 | 3.95 | ND |
| 2,4-Nonadienal, (E,E)- | Aldehydes | 3.08 | 0.20 | 3.20 | ND |
| Benzophenone | Benzenoids | 3.06 | 0.13 | 3.12 | SZ |
| Tridecane | Hydrocarbons | 3.05 | 0.15 | 3.24 | ND |
| Phenol, 4-pentyl- | Benzenoids | 2.98 | 0.14 | 2.91 | ND |
| 2,6-Lutidine | Heterocyclic_Compounds | 2.97 | 0.11 | 3.55 | KL |
| Pentadecanoic acid, ethyl ester | Esters | 2.96 | 0.10 | 3.85 | KL |
| Heptanal | Aldehydes | 2.83 | 0.15 | 3.57 | ND |
| 2,4-Heptadienal, (E,E)- |  | 2.53 | 0.11 | 3.15 | ND |
